# Machine Learning-Driven Radiomics for an Early-Stage Predictive Model of Nodal Upstaging in Thoracic Oncology

**DOI:** 10.3390/cancers18152470

**Published:** 2026-07-31

**Authors:** Ivan Lomangino, Giacomo Grisorio, Domenico Albano, Luca Vecchiarelli, Matteo Rota, Matteo Baldi, Letizia Perri, Mauro Roberto Benvenuti, Salvatore Grisanti, Francesco Bertagna

**Affiliations:** 1 Cardiothoracic Department, Thoracic Surgery Unit, Spedali Civili, 25123 Brescia, Italy; 2 Nuclear Medicine Department, Università degli Studi di Brescia, 25121 Brescia, Italy; domenico.albano@unibs.it (D.A.);; 3 Nuclear Medicine Department, ASST Spedali Civili of Brescia, 25123 Brescia, Italy; 4 Units of Biostatistics and Biomathematics and Bioinformatics, Department of Molecular and Translational Medicine, University of Brescia, 25121 Brescia, Italy; 5 Department of Thoracic Surgery, Università degli Studi di Padova, 35122 Padova, Italy; matteocesarebaldi@gmail.com; 6 Department of Thoracic Surgery, Università degli Studi di Modena e Reggio Emilia, 41121 Modena, Italy; 7 Medical Oncology, Department of Medical and Surgical Specialties, Radiological Sciences, and Public Health Medical, ASST-Spedali Civili, University of Brescia, 25121 Brescia, Italy

**Keywords:** lung cancer, machine learning, lymph node, metastasis

## Abstract

**Objectives:** Precise lymph node staging remains a cornerstone in the management of early-stage and locally advanced non-small cell lung cancer (NSCLC), directly influencing surgical planning and multimodal therapy. Despite the widespread use of 2-[18F]FDG PET/CT, occult nodal metastases frequently lead to unexpected upstaging after surgery, potentially affecting prognosis and therapeutic strategies. This study aimed to investigate whether radiomic features derived from preoperative PET/CT scans can predict nodal involvement in patients with early-stage lung cancer. **Methods:** A retrospective analysis was conducted on 124 patients with cT1N0 NSCLC who underwent 2-[18F]FDG PET/CT scans as part of the preoperative workup, followed by anatomical lung resection and systematic mediastinal lymph node dissection. Radiomic features were extracted from PET predictive of pathological nodal upstaging. **Results:** During the study period, 67 patients who underwent anatomical lung resection for early-stage lung cancer demonstrated unexpected nodal metastasis; a continuous series of 57 patients with the same clinical TMN was enrolled as a control group. Several radiomic parameters were significantly associated with nodal upstaging. According to variable importance (VIMP) analysis, metabolic tumor volume (MTV), total lesion glycolysis (TLG), run-length non-uniformity (RLNU), and gray-level non-uniformity (GLNU) emerged as the strongest predictors of lymph node involvement. **Conclusions:** Although the clinical utility of these findings remains to be validated, radiomic analysis of 2-[18F]FDG PET/CT imaging offers non-invasive biomarkers that may enhance the preoperative prediction of nodal involvement in early-stage NSCLC. Integrating radiomics into clinical workflows could improve surgical decision-making, refine patient selection, and reduce the incidence of unforeseen nodal upstaging.

## Simple Summary

This radiomic study evaluated whether radiomic features from preoperative 2-[18F]FDG PET/CT scans can predict occult lymph node metastases in early-stage non-small cell lung cancer (NSCLC). We enrolled patients with cT1N0 NSCLC with or without occult unexpected node metastasis after surgical resection. Radiomic features of the first and second level were evaluated and machine learning protocol was used to elaborate a predictive model. Both PET/CT and clinical protocols were identical for all patients and were performed at the same institution, providing robust, uniform data with consistent reconstruction parameters, thereby minimizing the data harmonization issue. Despite the inherent limitations of radiomics studies, we believe that the use of a homogeneous population, however small, can ensure the data uniformity necessary to identify a predictive model that, once refined, can be integrated into clinical practice to personalize the diagnostic pathway and, potentially, the surgical approach as well.

## 1. Introduction

Lung cancer remains the leading cause of cancer-related death worldwide despite being the second most frequently diagnosed malignancy. According to the most recent GLOBOCAN estimates, approximately 2.5 million new cases and 1.8 million deaths occur annually, accounting for nearly one in five cancer-related deaths globally. Although survival has improved, owing to screening programs and advances in multimodal treatment, prognosis remains highly dependent on disease stage at diagnosis [1]. The 5-year survival rate ranges from 5% in patients with metastatic disease to 57% when treated at an early stage [2]. Given its tendency to grow and spread to nearby tissues, lymph nodes (LNs), and distant sites, it is crucial to accurately determine the disease stage in planning comprehensive treatment. To date clinical staging involves imaging techniques such as computed tomography (CT) as well as 2-[18F]FDG PET/CT and invasive procedures like Endobrochial Ultrasound guided Transbronchial Needle Aspiration (EBUS-TBNA) and transcervical mediastinoscopy. The International Association for the Study of Lung Cancer uses the TNM classification to divide non-small cell lung cancer (NSCLC) into four stages [3]. Among these, stage III NSCLC (e.g., T1-2, N2, M0 and T3-4 N1/N2a M0 according to 9th TNM classification) is the most challenging: nodal involvement is a key point in planning multi-modal treatment, with neo-adiuvant and peri-operative chemo-immuno treatment that are by now clinical practice [4]. Thus, it is clear that providing stage-appropriate therapy and avoiding unnecessary surgery is critical. Once distant metastasis is ruled out, mediastinal lymph node involvement is the most significant prognostic factor [4]. Advancements in low-dose computed tomography screening have increased the precision of clinical staging for primary NSCLC at early stage [5]; however, a comprehensive assessment of disease spread and preoperative staging surely involves positron emission tomography. Therefore, establishing mandatory 2-[18F]FDG PET/CT preoperative workup could improve survival outcomes [6]. Currently, the most commonly used imaging technique for clinical lymph node (cN) staging is 2-fluorine-18-fluorodeoxyglucose positron emission tomography/computed 2-[18F]FDG PET/CT [7]. Numerous studies have suggested that 2-[18F]FDG PET/CT is superior to CT alone in evaluating mediastinal lymph node (MLN) metastasis in NSCLC (sensitivity: 79–85% vs. 57–68%) [8,9,10]. This superiority has been demonstrated in both staging and predicting upstaging in adenocarcinomas, facilitating stage-appropriate treatment, eliminating unnecessary invasive procedures, and optimizing multimodal treatment algorithms [11]. However, when comparing radiological versus pathological staging, unexpected pathological lymph nodes are still quite uncommon. Nodal upstaging occurs when previously undetected lymph node metastases are identified through pathological evaluation after surgical treatment for NSCLC. In early-stage NSCLC, this rate ranges from 4.8% to 24.6%, depending on factors such as the accuracy of preoperative staging, tumor size and location, and extension of lymphadenectomy. Unexpected lymph node disease should be considered a failure of preoperative disease staging and a potential risk for incorrect surgical decisions. Therefore, invasive mediastinal exploration and pathological evaluation remain necessary [12].

Radiomics is an expanding and promising field of contemporary medicine, useful for investigating tumor properties that go beyond visual interpretation or traditional imaging, such as unseen nodal disease. Radiomics involves extracting and analyzing quantitative image features from medical images (texture analysis) using mathematical and machine learning methods (e.g., random forests) to correlate these features with the biological or clinical behavior of the lesion [13]. The advent of the ‘big data’ era opens up the possibility that radiomics could replace invasive procedures for lymph node staging. Radiomics has been assessed in several studies [14,15], and although the results are promising, the diagnostic value remains unclear.

Several studies demonstrate the utility of radiomics in predicting metastasis or prognostic value—particularly in upper gastrointestinal cancers, in addition to lung cancer [16]—using supervised algorithms including Random Forest, Support Vector Machines, Extreme Gradient Boosting and deep learning architectures. Particularly, they have attempted to explore the predictive capacity of radiomic features associated with the primary tumor in lymph node involvement, with conflicting results [17,18,19]. However, it seems that increasingly radiomic variables could be used as predictive markers for lymph node metastasis [19,20,21,22].

This study aimed to identify predictive parameters in texture analysis of 2-[18F]FDG PET/CT imaging to assess lymph node upstaging in NSCLC using machine learning analysis.

## 2. Materials and Methods

### 2.1. Patient Characteristics

**Figure 1:**
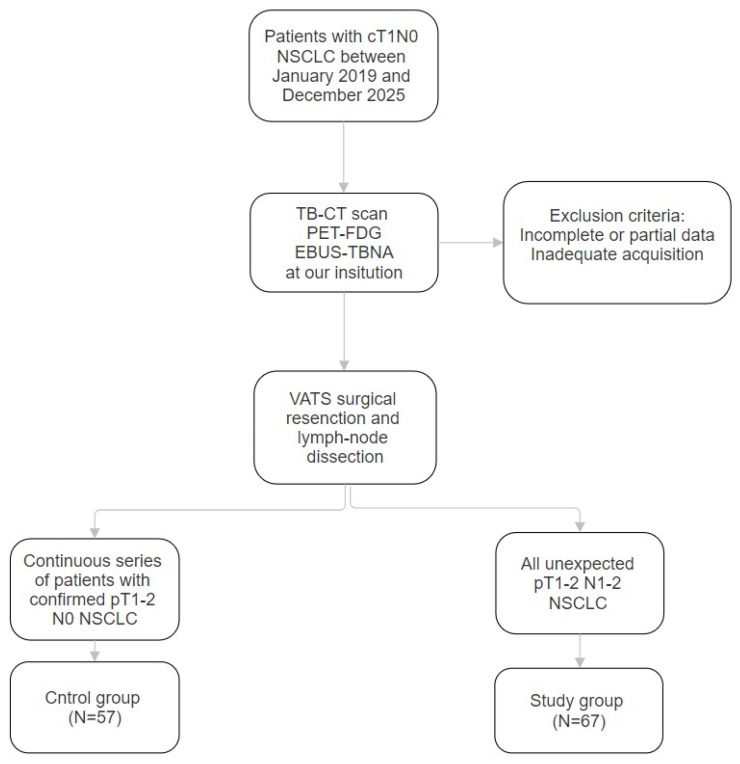
CONSORT representation of patient’s selection.

This retrospective, single-center study included patients diagnosed with NSCLC—specifically adenocarcinoma and squamous cell carcinoma—who underwent surgical treatment. We evaluated all patients with clinical T1N0 NSCLC according to the 8th edition of the TNM classification who underwent surgery at our center during a five-year period from January 2019 to January 2024 and experienced nodal upstaging. These patients were compared to a consecutive series of 57 patients with clinical T1N0 NSCLC treated surgically from January 2024 onwards who did not show nodal upstaging on pathological staging (Figure 1).

Inclusion criteria encompassed early-stage NSCLC (cT1a-T1b-T1c N0), defined by a preoperative 2-[18F]FDG PET/CT scan performed within two months prior to surgery at the Nuclear Medicine Department of Spedali Civili di Brescia. Every patient underwent mediastinal staging via EBUS-TBNA without evidence of hilar or mediastinal disease. All patients received anatomical lung resection and systematic hilar-mediastinal lymphadenectomy using a Video-Assisted Thoracoscopic Surgery (VATS) biportal technique.

Exclusion criteria included a history of neoadjuvant therapy, prior cancer diagnosis, cancer recurrence, or synchronous/metachronous neoplasms.

The baseline characteristics between the groups included age, sex, SUV max, tumor size, histology and distance from the visceral pleura. We then analyzed which 2-[18F]FDG PET/CT features could be predictive of lymph node upstaging in the comparison between the two groups. Patients for whom it was not possible to fully extract all radiomic features were excluded from the study.

Ethical approval for this study was obtained from the Ethical committee Lombardia 6 (NP6781, 12 January 2025), a written informed consent for participation was signed by all patients.

### 2.2. PET/CT Acquisition and Interpretation

2-[18F]FDG PET/CT scanning was performed according to international guidelines.

In order to perform the 2-[18F]FDG PET/CT scan, 3.5–4.5 Mbq/kg of [18F]FDG were intravenously injected into the patients, and before image acquisition they were instructed to void. Patients fasted for at least 6 h and need to have a glucose plasma level below 150 mg/dL (mean: 120, range: 69–149). No other contrast agent or intestinal preparation were used.

Images were acquired 60 min after injection, from the apex to the mid-thigh on two different PET/CT tomographs. The first one (scanner 1) was a Discovery 690 PET/CT (General Electric Company, Milwaukee, WI, USA) while the second (scanner 2) was a Discovery STE PET/CT (General Electric Company, Milwaukee, WI, USA). On both, standard acquisition parameters (CT: 80 mA, 120 kV without contrast; 2.5–4 min per bed PET-step, axial width 15 cm) and standard reconstruction parameters were used (256 × 256 matrix and 60 cm field of view). Furthermore, scanner 1 had LYSO (cerium-doped lutetium yttrium oxyorthosilicate) scintillator crystals with a decay time of 45 ns, while scanner 2 had BGO (bismuth germanate) scintillator crystals with a decay time of 300 ns. Scanners were not harmonized with a cross-calibration program and all PET/CT scans were acquired at free-breath, instructing the patients to have regular breathing. For anatomical correlation and to perform attenuation correction, a low-dose CT at free-breathing and without contrast agent was acquired for both the scanners. More in detail, CT acquisition parameters for scanner 1 were: 120 kV, fixed tube current ≈ 60 mAs (40–100 mAs), 64 slices × 3.75 mm and 3.27 mm interval, pitch 0.984:1, tube rotation 0.5 s. CT acquisition parameters for scanner 2 were: 120 kV, fixed tube current ≈ 73 mAs (40–160 mAs), 4 slices × 3.75 mm and 3.27 mm interval, pitch 1.5:1, tube rotation 0.8 s. Furthermore, on scanner 1, time of flight (TOF) and a point spread function (PSF) algorithm were used for the reconstruction of images, with a filter cut-off of 5 mm, 18 subsets and 3 iterations. Moreover, on scanner 2, an ordered subset expectation maximization (OSEM) algorithm with a filter cut-off of 5 mm, 21 subsets and 2 iterations was used.

2-[18F]FDG PET/CT images were visually and semiquantitatively analyzed by a nuclear physician with more than 10 years of experience, and every focal tracer uptake deviating from physiological distribution and background was regarded as suggestive of disease localization.

### 2.3. Radiomics Features Extraction

LIFEx 7.7 software (http://www.lifexsoft.org; accessed on 1 May 2025) was used to segment 2-[18F]FDG PET/CT [23]. VOI was draw on 18F-FDG PET/CT images corrected for attenuation, using an SUV-based automated contouring program with an isocounter threshold method based on 41% of the SUVmax.

Several radiomic features were extracted from the 2-[18F]FDG PET/CT images, all regarding tumor (T) characteristic, divided as follows:

- First-order statistics: SUV-related, metabolic tumor volume (MTV), Total Lesion Glycolysis (TLG), histogram-related and shape-related;
- Second-order statistics: gray-level co-occurrence matrix-related (GLCM), gray-level run length matrix-related (GLRLM), neighborhood gray-level different matrix-related (NGLDM) and gray-level zone length matrix-related, (GLZLM).

A complete glossary of the radiomics features examined and radiomics abbreviations are listed in Table S1.

LIFEx calculates random forest (RFS) only for VOIs of at least 64 voxels. These measurements were performed by a reader with experience in this kind of analysis.

### 2.4. Machine Learning and Statistical Analysis

Patients’ baseline and tumor characteristics were summarized by using frequencies and percentages for categorical variables, and medians with interquartile ranges (IQRs) for continuous variables. The chi-square test for categorical variables and the Wilcoxon rank-sum test (Mann–Whitney U test) for continuous variables were used to compare cases and controls.

Four machine learning algorithms—logistic regression, penalized logistic regression through lasso, support vector machine (SVM) with linear Kernel and random forest (RF)—were employed to develop predictive models for lymph node upstaging in NSCLC. Due to the limited sample size, repeated internal cross-validation was used to assess models’ performance. Specifically, the dataset was split into five folds, and the process was repeated 10 times, resulting in 50 different held-out sets for model evaluation.

The predictive performance of the models was assessed using the receiver operating characteristic (ROC) curve and the area under the curve (AUC), which quantified the effectiveness of radiomic features in predicting lymph node upstaging in NSCLC [24]. In addition, specificity, sensitivity, positive predictive value (PPV), and negative predictive value (NPV) were also calculated.

The penalized logistic regression model was implemented using the Lasso algorithm, with the lambda parameter selected as the one that maximized the AUC. For the SVM and RF algorithms, hyperparameter tuning was carried out for the misclassification penalty parameter using a grid of values ranging from 0 to 2, selecting the value that yielded the highest accuracy.

For feature selection and predictive modeling, a random forest algorithm with hyperparameter tuning was employed.

The model was optimized using random grid search combined with 5-fold repeated (n = 10) cross-validation. Each iteration was evaluated based on accuracy, and the best hyperparameters for the random forest model were determined accordingly.

To assess feature relevance, the Variable Importance (VIMP) measure was computed for each feature.

Features with VIMP > 40 were considered significant predictors. The final predictive model was selected based on the highest NPV, as the PPV of 2-[18F]FDG PET/CT in assessing nodal disease was considered sub-optimal due to the challenges in interpretation arising from a variety of inflammatory and reactive conditions that can lead to increased FDG uptake and imaging features mimicking malignancy.

The predictive performance of the model was evaluated using the receiver operating characteristic (ROC) curve and the area under the curve (AUC), which measured the effectiveness of the 2-[18F]FDG PET/CT radiomic features in predicting lymph node upstaging in NSCLC [25].

The R package “caret” was used for model training. The significance level was set at α = 0.05, with *p* ≤ 0.05 considered statistically significant.

## 3. Results

### 3.1. Patient Demographics and Tumor Characteristics

Table 1 summarizes the characteristics of the population and the tumor, showing no statistically significant differences between the two groups compared.

The study group consisted of 31 female and 36 male patients with a median age of 69 years (interquartile range 64–73). The control group included 24 female and 33 male patients, with a median age of 70 years (interquartile range 64–74). No significant differences were observed between groups regarding gender or age (Wilcoxon rank sum test *p*-value 0.6 and 0.7, respectively.

Two-thirds of the cases were adenocarcinoma, although higher preoperative Stardardized Uptake Value (SUV) max values of the primary tumor were more frequently associated with squamous cell carcinoma. SUV max was 10.4 in study group and 6.2 in control group (*p* < 0.001).

The tumor size, expressed as the T diameter, was compared between the two groups, showing no significant differences (*p* = 0.5). We also investigated the distance of the neoplasm from the closest visceral pleural plane to the lesion in order to determine whether lesions that were more central or closer to the lymphatic plane might have a greater tendency toward nodal upstaging. Our concern was that the position of the neoplasm could influence lymph node metastasis and serve as a bias in our study. The average distance from the visceral pleura—including the fissures, expressed in mm—was 4.47 mm in the case group and 5.9 mm in the control group, a difference that was not statistically significant (*p* = 0.3).

### 3.2. Comparison of the Radiomic PET Variables

Significant differences were observed between the two groups regarding radiomic variable; 2-[18F]FDG PET/CT features revealed that patients with higher SUVmax values demonstrated a strong association with nodal upstaging (case group: 10.4 vs. control group: 6.27; *p* < 0.001) (Table 2).

**Figure 2:**
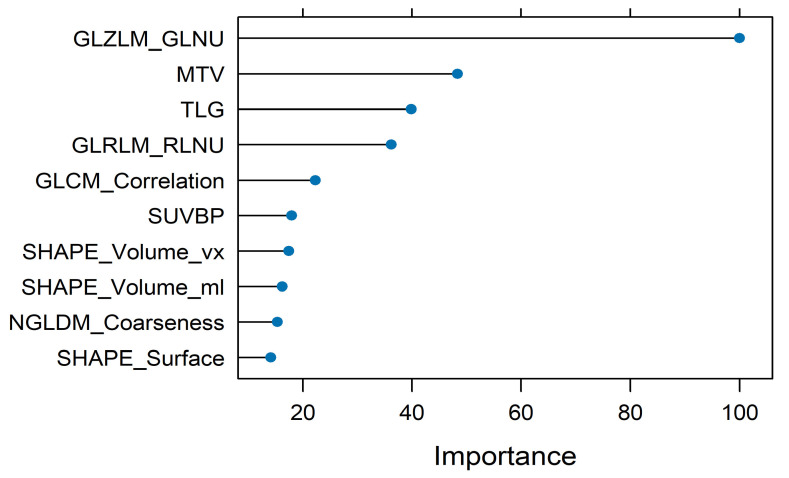
Visual representation of variable importance calculation. Features with a VIMP > 40 were selected among the statistically significant ones (blue dots over 40).

The analysis between the two groups, particularly the comparison of the first-, second-, and third-order PET features, revealed remarkable, statistically significant differences between the two groups. PET features were considered significant if they had a Variable Importance Measure (VIMP) of at least 40, as visually described in Figure 2. The most predictive PET-derived radiomic features included GLNU, Metabolic Tumor Volume (MTV), Total Lesion Glycolysis (TLG), and Run Length Non-Uniformity (RLNU). The gray-level zone length matrix (GLNU) exhibited the highest VIMP score and demonstrated robust predictive capabilities.

Patients with early-stage NSCLC undergoing surgical treatment were evaluated to predict nodal occult metastasis. Radiomic features from FDG PET/CT were compared between patient with and without occult nodal metastasis. Machine learning algorithms were used to build a predictive model with 74% accuracy and based on the feature with the greatest variable importance (MTV, TLG, RLNU and GLNU).

**Figure 3:**
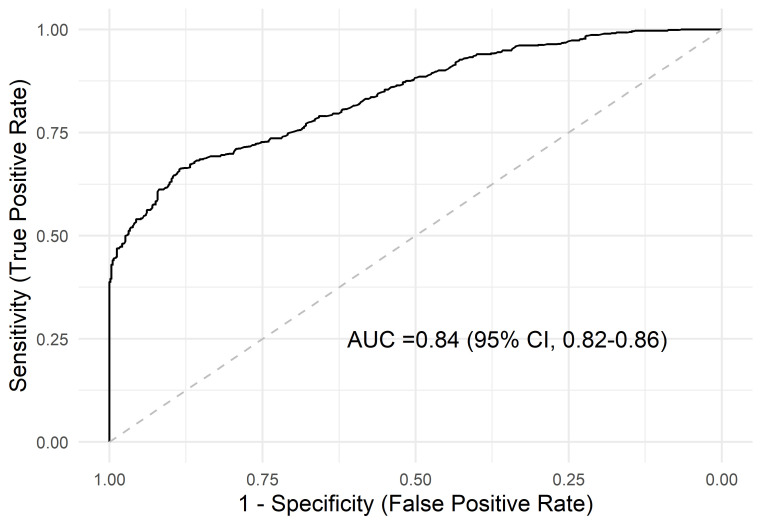
Cross validation of model’s performance by AUC.

Cross-validated classification analysis yielded the following performance metrics for predicting nodal upstaging:

- AUC-ROC: 0.84 (Figure 3);
- Sensitivity: 72%;
- Specificity: 77%;
- Overall Accuracy: 74%.

These findings underscore the potential of radiomic texture features in refining preoperative staging accuracy.

## 4. Discussion

Our study has some limitations. As a retrospective and single-center study, we acknowledge the potential risk of selection bias. The careful selection of our study cohort led to a relatively small sample size, which, although consistent with previous studies in the literature, limits the robustness of the statistical analysis. Data heterogeneity is one of the main limitations of radiomics studies such as ours; using the same scanner with a standardized acquisition and reconstruction protocol would limit this heterogeneity. We believe, however, that patient selection based on definitive histological confirmation of lymph node involvement is a strength of our study. Furthermore, all patients underwent the same diagnostic evaluation via EBUS-TBNA at the same institution, and all evaluated 2-[18F]FDG PET/CT scans were performed within a timeframe that overlapped with the surgical procedure; clinical evaluation and surgical procedures were performed at the same institution by a multidisciplinary team. The design of this study ensures data consistency and limits acquisition variability, which remains a significant issue even in more recent studies, especially multicenter ones [19,20,21].

The lack of external validation is the main methodological limitation of this study; our results represent only the first step toward a broader application of radiomics. More in-depth and extensive studies are needed to enable the implementation of radiomics findings in clinical practice; in particular, due to the great methodological variability in radiomics studies, it is currently not possible to recommend a specific reconstruction or segmentation method, which limits the ability to standardize studies and make them fully comparable [26].

We believe that the design of the study allows us to reach interesting results. Nearly all first-, second-, and third-order variables observed showed a statistically significant difference between the study group and the control group, highlighting that the metabolic activity of the neoplasm in lymph node upstaging is at least different from that of a neoplasm without lymph node involvement. In the study group, the mean SUVmax value was higher than the control group, as Li et al. [27] previously demonstrated a strong association between SUVmax and lymph node upstaging, identifying an SUVmax value greater than 4.3 as a predictor of lymph node involvement. Miyasaka et al. [28] not only confirms this association but also correlates SUVmax with prognosis, where there is an inversely proportional relationship between SUV and the benignity of the prognosis, especially above a value of 10. Wu et al. [29] highlighted that, as in our study, for tumors with a diameter smaller than 2 cm, SUVmax is one of the major predictive factors for lymph node metastasis. Tapias et al. [6] demonstrated that SUVmax was correlated with a higher risk of lymph node involvement, particularly as N1 in station 11.

Regarding the values obtained with radiomics, the statistical analysis allowed us to identify four parameters that in our group of patients were more strongly predictive of lymph node upstaging. We applied machine learning algorithms to obtain different models of predictive analysis. Surprisingly, all the results were remarkable, but we chose random forest as it was a more descriptive framework.

As demonstrated in other studies, there is an association between features such as MTV and TLG and lymph node disease. Okumus et al. [5] highlighted that an increase in MTV was present in patients with lymph node upstaging. Kim et al. [30] studied the prognostic value of MTV and TLG, finding them to be significantly predictive of survival and disease recurrence.

The gray-level zone length matrix (GLZLM) is a regional texture feature that provides information on the size of homogeneous zones for each gray level in three dimensions. Gray-level non-uniformity (GLNU) is a measure of the similarity of gray-level values throughout the image. This feature was found to be the most relevant predictor of lymph node upstaging in our study using the RF model; its value increases with the heterogeneity of the lesion. Therefore, in texture analysis, greater heterogeneity of the lesion is associated with greater tumor aggressiveness in terms of lymph node metastasis. To date, this same value has been found to predict neoplastic aggressiveness in other studies, but not yet for lung cancer.

It is interesting to observe that 2-[18F]FDG PET/CT—already an essential examination for lung cancer staging, indispensable as a preoperative test, and now commonly used in the work-up of patients with resectable lung neoplasms—holds predictive potential that has so far been underutilized. The prospect of refining the diagnostic capabilities of 2-[18F]FDG PET/CT through radiomics—independently of the operator—to provide further information on tumor aggressiveness in local lymph nodes—and thus offer valuable support in identifying early-stage tumors at higher risk of metastasis—is truly intriguing. Recent advancements have focused on integrating multimodal datasets, such as genomics and neuroimaging, to improve diagnostic accuracy in different fields [31]; this approach is the perfect embodiment of advanced computational capabilities applied to creating new clinical tools. The future applications of PET/CT radiomics are particularly interesting because they could enable—through machine learning mechanisms and thanks to ever-increasing computing power—the mapping of hilar and mediastinal lymph nodes at higher risk of tumor involvement [32].

## 5. Conclusions

The 2-[18F]FDG PET/CT radiomic model has the ability to enhance its reliability, particularly by correlating certain features with tumor aggressiveness or, more specifically, with the tendency for lymph node upstaging. MTV, TLG, and GLRLM_RLNU have proven to be excellent predictors of lymph node involvement, while GLZLM_GLNU emerged as the strongest predictor.

Looking ahead, future studies could explore the correlation between the radiomic features of the T stage and those of the N stage that underwent upstaging, and assess the predictive value of radiomics in relation to mediastinal recurrence and overall survival.

## Figures and Tables

**Table 1:** Baseline characteristics of control and study group.

	Study Group (*n* = 67) ^1^	Control Group (*n* = 57) ^1^	*p*-Value ^2^
Sex			0.6
F	31 (47%)	24 (42%)	
M	36 (53%)	33 (58%)	
Age	69 (64, 73)	70 (61, 74)	0.7
T Dimensions (mm)	18.46	18.22	0.5
T Distance from pleura (mm)	4.47	5.9	0.3
SUV max	10.4	6.27	<0.001

^1^ *n* (%); Median (IQR), ^2^ Pearson’s Chi-squared test; Wilcoxon rank sum test.

**Table 2:** All the radiomic characteristics evaluated, a large part of them result as statistically significant.

Characteristic	Study Group (n = 67)	Control Group (n = 57)	*p*-Value	Q-Value
SUV max	9.6 (5.3–14.8)	4.7 (2.9–7.8)	<0.001	<0.001
SUV mean	5.5 (3.4–10.6)	2.8 (1.7–4.7)	<0.001	<0.001
MTV	8 (4–22)	3 (2–5)	<0.001	<0.001
TLG	47 (16–202)	8 (4.24)	<0.001	<0.001
DISCRETIZED_HISTO_ Skewness	2.30 (1.41–3.46)	3.46 (2.60–4.39)	<0.001	<0.001
DISCRETIZED_HISTO_ Kurtosis	7 (3–14)	14 (10–22)	<0.001	<0.001
DISCRETIZED_HISTO_ Excess_Kurtosis	5 (0–11)	11 (6.19)	<0.001	<0.001
DISCRETIZED_HISTO_ Energy	0.09 (0.05–0.14)	0.16 (0.09–0.25)	<0.001	<0.001
SHAPE_Volume_ml	8 (4.21)	3 (2–6)	<0.001	<0.001
SHAPE_Volume_vx	345 (155–903)	134 (79–235)	<0.001	<0.001
SHAPE_Sphericity	0.85 (0.76–0.89)	0.87 (0.83–0.92)	0.007	0.01
SHAPE_Surface	2.465 (1.321–4.592)	1.203 (850–1.883)	<0.001	<0.001
SHAPE_Compacity	3.57 (2.76–4.27)	2.57 (2.32–3.21)	<0.001	<0.001
SHAPE_Homogeneity	0.34 (0.28–0.42)	0.44 (0.29–0.55)	0.058	0.068
GLCM_Energy	0.01 (0.01–0.03)	0.03 (0.01–0.06)	<0.001	<0.001
GLCM_Contrast	18 (9–40)	6 (2–6)	0.010	0.013
GLCM_Correlation	0.47 (0.34–0.56)	0.31 (0.22–0.44)	<0.001	<0.001
GLCM_Entropy_log10	2.07 (1.70–2.41)	1.65 (1.36–1.92)	<0.001	<0.001
GLCM_Entropy_log2	6.88 (5.64–7.99)	5.41 (4.48–6.28)	<0.001	<0.001
GLCM_dissimilarity	3.45 (2.38–5.10)	1.95 (1.11–4.12)	0.010	0.013
GLRLM_SRE	0.95 (0.92–0.96)	0.92 (0.87–0.97)	0.2	0.2
GLRLM_LRE	1.25 (1.18–1.41)	1.35 (1.13–1.70)	0.4	0.4
GLRLM_LGRE	0.003 (0.001–0.010)	0.013 (0.005–0.033)	<0.001	<0.001
GLRLM_HGRE	352 (122–1.279)	94 (39–263)	<0.001	<0.001
GLRLM_SRLGE	0.003 (0.001–0.009)	0.012 (0.004–0.027)	<0.001	<0.001
GLRLM_SRHGE	332 (114–1.233)	86 (34–250)	<0.001	<0.001
GLRLM_LRLGE	0.00 (0.00–0.001)	0.02 (0.01–0.07)	<0.001	<0.001
GLRLM_LRHGE	450 (196–1488)	139 (70–316)	<0.001	<0.001
GLRLM_GLNU	30 (15–55)	17 (8–34)	0.005	0.008
GLRLM_RLNU	261 (126–723)	83 (60–163)	<0.001	<0.001
GLRLM_RP	0.93 (0.89–0.95)	0.90 (0.81–0.96)	0.3	0.3
NGLDM_Coarseness	0.017 (0.008–0.029)	0.036 (0.023–0.050)	<0.001	<0.001
NGLDM_Contrast	0.22 (0.12–0.37)	0.16 (0.07–0.37)	0.2	0.2
NGLDM_Busyness	0.32 (0.17–0.60)	0.39 (0.16–0.90)	0.3	0.3
GLZLM_SZE	0.57 (0.48–0.66)	0.42 (0.31–0.64)	0.013	0.017
GLZLM_LZE	25 (10–165)	75 (5–470)	0.5	0.5
GLZLM_LGZE	0.003 (0.001–0.010)	0.012 (0.005–0.034)	<0.001	<0.001
GLZLM_HGZE	375 (131–1171)	108 (45–384)	<0.001	<0.001
GLZLM_SZLGE	0.002 (0.001–0.004)	0.004 (0.001–0.007)	0.006	0.008
GLZLM_SZHGE	216 (56–747)	39 (10–179)	<0.001	<0.001
GLZLM_LZLGE	0 (0–1)	2 (0–17)	0.015	0.018
GLZLM_LZHGE	11,480 (5548–51,545)	5360 (2378–20,938)	0.008	0.011

## Data Availability

The original contributions presented in this study are included in the article/Supplementary Material. Further inquiries can be directed to the corresponding author.

## Supplementary Materials

The following supporting information can be downloaded at: https://www.mdpi.com/article/10.3390/cancers18152470/s1, Table S1: a comprehensive glossary of radiomics terms and features.

## Abbreviations

The following abbreviations are used in this manuscript:

2-[18F]FDG PET/CT	fluorine-18-fluorodeoxyglucose positron emission tomography/computed
CT	computed tomography
AUC	area under the curve
EBUS-TBNA	Endobrochial Ultrasound guided Transbronchial Needle Aspiration
GLCM	Gray-level co-occurrence matrix, related
GLNU	gray-level zone non-uniformity
GLRLM	gray-level run length matrix
GLZLM	gray-level zone length matrix
MLN	mediastinal lymph node
MTV	metabolic tumor volume
NGLDM	neighborhood gray-level different matrix-related
NPV	negative prediction value
NSCLC	non-small cell lung cancer
PPV	positive prediction value
OSEM	ordered subset expectation maximization
PSF	point spread function
ROC	receiver operating characteristic
RF	random forest
RNLU	Run Length Non-Uniformity
SUV	Stardardize Uptake Value
SVM	support vector machine
TLG	Total Lesion Glycolysis
TOF	time of flight
VATS	Video-Assisted Thoracoscopic Surgery
VIMP	Variable Importance
VOI	volume of interest
