# Supplementary material for: Machine Learning-Driven Radiomics for an Early-Stage Predictive Model of Nodal Upstaging in Thoracic Oncology
Source: Cancers (Basel). 2026 Jul 31;18(15):2470. doi: 10.3390/cancers18152470 (PMC13463928; doi:10.3390/cancers18152470)
Supplement: Supplementary file 1 [file cancers-18-02470-s001.zip › cancers-4386422-supplementary.pdf]

cancers

MDPI

Table S1. A comprehensive glossary of radiomics terms and features.

Name	Description
**Volume_ml**	Volume of the lesion in milliliters.
**Volume_vx**	Volume of the lesion in number of voxels.
**Sphericity**	The closer the value is to 1, the closer the lesion is to the spherical shape
**Surface**	Total surface of the lesion
**Compacity**	Volume-to-surface ratio, how compact or regular the shape of the lesion is compared to a perfect sphere.
**Homogeneity**	Evaluates the similarity of adjacent voxels—a higher value suggest a more uniform tissue.
**SUV**	Standard Uptake Value. The quantitative measure of the metabolic activity of a tissue, based on the quantity of radioactive tracer absorbed in an area.
**SUV max**	Maximal value of tracer uptake in a voxel. It indicates the most intense metabolic activity of the lesion.
**SUV mean**	It indicates the mean metabolic activity of the lesion.
**MTV**	Metabolic Tumor Volume. Calculated considering the voxels with a SUV superior to a defined value.
**TLG**	Total Lesion Glycolysis, the product of MTV and SUV mean. It expresses the total metabolic activity of the lesion.
**Skewness**	It measures the asymmetry in distribution of SUV values.
**Kurtosis**	It expresses the narrowness in distribution. Higher values are index of a distribution with peaks.
**Excess_Kurtosis**	Difference in kurtosis between the observed one and a normal distribution. Positive values suggest higher peaks.
**Energy**	Sum of squared SUVs. It measures the magnitude of voxel values Higher values show more uniformity in texture.
**Contrast**	Measure of the local gray-level variation. Higher values suggest major contrast.
**Correlation**	The correlation of adjacent pixel. The closer the value is to 1 or -1, the stronger the correlation.
**Entropy_log10**	It measures image complexity; the higher the value, the bigger the disorder.
**Entropy_log2**	The same as before but the logarithm as a base of 2.
**Dissimilarity**	The difference in pixel couples; the higher the value, the bigger the difference.
**SRE**	Short Run Emphasis. It measures the predominance of short pixel sequences with the same gray level.
**LRE**	Long Run Emphasis. It measures the predominance of long pixel sequences with the same gray level.
**LGRE**	Low Gray Level Run Emphasis. It emphasizes the sequences with a low gray level.
**HGRE**	High Gray Level Run Emphasis. It emphasizes the sequences with a high gray level.
**SRLGE**	Short Run Low Gray Level Emphasis. It measures the presence of short sequences with a low gray level.

 https://doi.org/10.3390/cancers18152470

**SRHGE**	Short Run High Gray Level Emphasis. It measures the presence of short sequences with a high gray level.
**LRLGE**	Long Run Low Gray Level Emphasis. It measures the presence of short sequences with a low gray level.
**LRHGE**	Long Run High Gray Level Emphasis. It measures the presence of short sequences with a high gray level.
**GLNU**	Gray Level Non-Uniformity. It measures the variability of gray level.
**RLNU**	Run Length Non-Uniformity. It measures the variability of sequence length.
**RP**	Run Percentage. The total number of sequences to total number of voxel ratio. It is the sequences' density index.
**Coarseness**	It measures the uniformity of textures. The higher the value, the more uniform the texture.
**Contrast**	Evaluates the variation in intensity between a voxel and its neighbors. Higher values indicate a greater variation.
**Busyness**	It measures how quickly gray levels change between adjacent voxels; higher values indicate more complex textures.
**SZE**	Short Zone Emphasis; measures the predominance of small areas with the same gray level.
**LZE**	Large Zone Emphasis; measures the predominance of big areas with the same gray level.
**LGZE**	Low Gray Level Zone Emphasis; emphasizes areas with low gray levels.
**HGZE**	High Gray Level Zone Emphasis; emphasizes areas with high gray levels.
**SZLGE**	Short Zone Low Gray Level Emphasis; measures the presence of small zones with low gray levels.
**SZHGE**	Short Zone High Gray Level Emphasis; measures the presence of small zones with high gray levels.
